# Species Identification of Birds Nasal *Trichobilharzia* in Sari, North of Iran

**Published:** 2012

**Authors:** SH Maleki, A Athari, A Haghighi, N Taghipour, SH Gohardehi, S Seyyed Tabaei

**Affiliations:** 1 Dept. of Parasitology, School of Medicine, Shahid Beheshti University, M. C., Tehran, Iran; 2 Dept. of Parasitology, School of Medicine, Mazandaran University, M. C., Sari, Iran

**Keywords:** *Trichobilharzia regenti*, Cercarial Dermatitis, PCR, Iran, Anatid

## Abstract

**Background:**

Cercarial dermatitis is known as an endemic parasitic disease in North of Iran, a hypersensitive skin reaction to the penetration of nonhuman schistosome larvae into human skin. In recent studies in this region, final and intermediate hosts were recognized and *Trichobilharzia* was identified as the main causative agent of cercarial dermatitis in this region, but to date the parasite species haven't been identified. Therefore this study was performed to species identification of nasal *Trichobilharzia* in infected birds for the first time.

**Methods:**

A total of 45 *Anas clypeata* birds identified as final host, were collected from Sari in North of Iran and infected nasal tissues analyzed using molecular techniques. Genomic DNA was isolated by phenol/chloroform extraction method and ITS region of rDNA were amplified with specific primers its5Trem and its4Trem, then sequenced area were compared with existing records in GenBank.

**Results:**

Twelve samples were infected with *Trichobilharzia* and results of PCR reaction indicated that all of them belonged to *T. regenti*. The sequence alignment of present work isolates and those deposited in GenBank showed differences in nucleotide sequences of repeat region in ITS1.

**Conclusion:**

*Trichobilharzia regenti* is the most frequent parasite of Anatid birds in North of Iran. This corresponds to the distribution of this parasite along the flyway of migratory birds, which annually migrate from Siberia and northern countries of Caspian Sea to wintering areas in southern regions of it.

## Introduction

*Trichobilharzia* is the largest genus within the family Schistosomatidae, covering over 40 species of avian parasites (1). Cercaria of this genus are the main causative agent of cercarial dermatitis (2), an allergic response to the penetration of schistosome larvae into non-specific skin (3). Experimental infections have shown that *Trichobilharzia* species can not mature in incompatible hosts, however parasites may migrate throughout the viscera and the nervous system of mammals and as a consequence might cause some injuries, therefore a precise determination of species is valuable not only from the biological point but also as a tool to predict health risks (1).

Generally, the species of *Trichobilharzia* can be divided into two main groups: visceral schistosomes and nasal schistosomes, as there are lots of difficulties for species determination by using morphologic characterization of parasite, in recent years, species identification are assessed by sequence analysis of DNA (1, 4).

Cercarial dermatitis is an endemic disease among rice planters in North and South of Iran (5–8), and in previous studies on definite and intermediate hosts, *Trichobilharzia* has been morphologically determined and introduced as a responsible genus for the majority of swimmers itch cases in both area (6–8). Surprisingly since the genus description which has been nearly 20 years ago, species identity remained unknown in the affected areas.

Sari, the capital of Mazandaran Province, is located in North of Iran, the presence of intermediate snails and thousands of birds that migrate to Iran's natural habitats annually complete the life cycle of the parasite (8). According to the results of previous studies in this area, the prevalence rate of infection in definite hosts has been 18.1% among the total of 138 aquatic birds and the most infected birds belonged to *Anas clypeata* (76.9%) (8). So in this study, Anatid birds belong to this species were collected during winter from villages in the vicinity of Sari and dissected.

The aim of our present work was to determine the species of the nasal *Trichobilharzia* found in their nasal tissues using molecular methods.

## Materials and Methods

### Samples

A total of 45 migrating ducks belonging to *Anas clypeata* were collected from Sari the capital of Mazandaran Province which during our previous study had been performed (8).

### Parasitological and microscopic examination

The nasal area of each bird was cut open with two deep incisions in each side of the beak, then two pieces of nasal mucosa removed and ethanol preserved for molecular analysis.

Five out of 45 samples were placed in Petri dish, dissected with fine needles and examined for presence of nasal schistosomes under the dissecting microscope.

### DNA extraction

Genomic DNA was isolated by phenol/chloroform extraction method from eggs, fragments of adults and nasal tissues.

### PCR

ITS region was amplified with specific primers its5Trem (5’-GGAAGTAAAAGTCGTAACAAGG-3’) complementary to the conserved region at the 3’ end of the 18S rRNA gene and its4Trem (5’-TCCTCCGCTTATTGAT-ATGC-3’) complementary to the conserved region at the 5’ end of the 28S rRNA gene.

PCR was performed in reaction volume of 30µl.The reaction mixture contained 1X PCR buffer, 1.5 mM MgCl2, 0.3 mM dNTP, 1.25 U *Taq* polymerase (Cinnagen, Iran), 1µl of the DNA template and 1.5 µM of each forward and reverse primers.

The PCR products were visualized by gel electrophoresis using 1.5% agarose, and the results were recorded on a UV gel documentation system (Cinnagen Ltd, Tehran, Iran). Purified PCR products were directly sequenced with forward primer its5Trem.

## Results

**Fig. 1:**
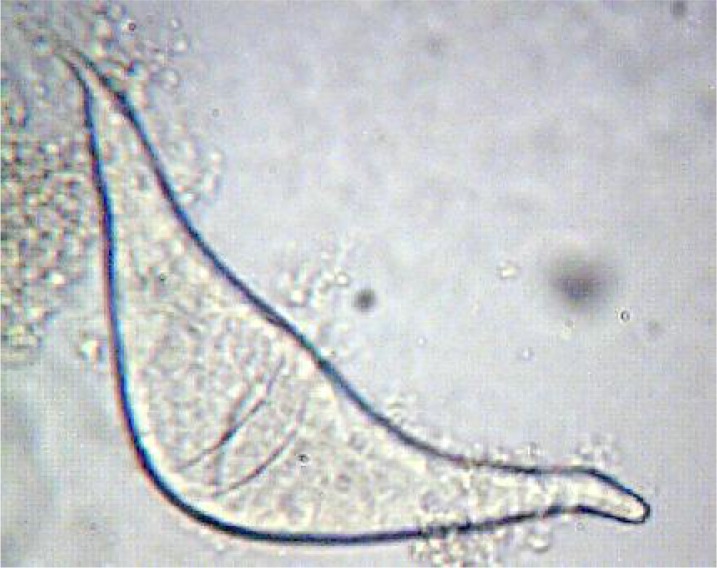
Egg of nasal *Trichobilharzia* spp. isolated from *Anas clypeata* (Original)

**Fig. 2:**
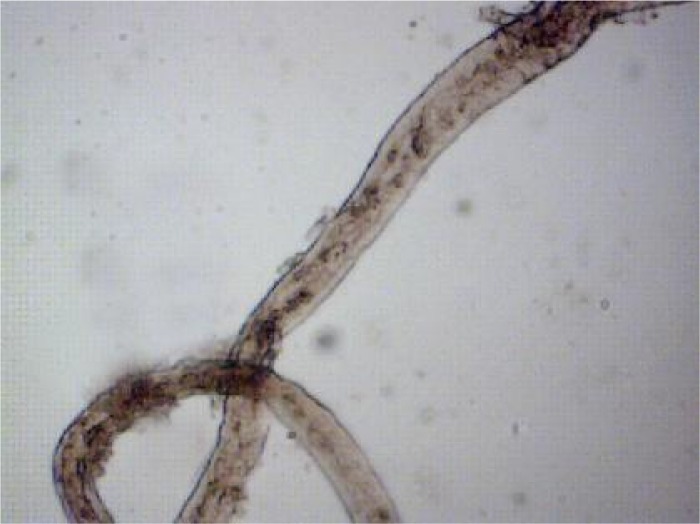
Adult of nasal *Trichobilharzia* spp. isolated from *Anas clypeata* (Original)

Eggs (Fig. 1) and fragments of adults (Fig. 2) isolated in microscopic examination from 5 samples, and based on their morphology, they were all determined as *Trichobilharzia* spp.

**Fig. 3:**
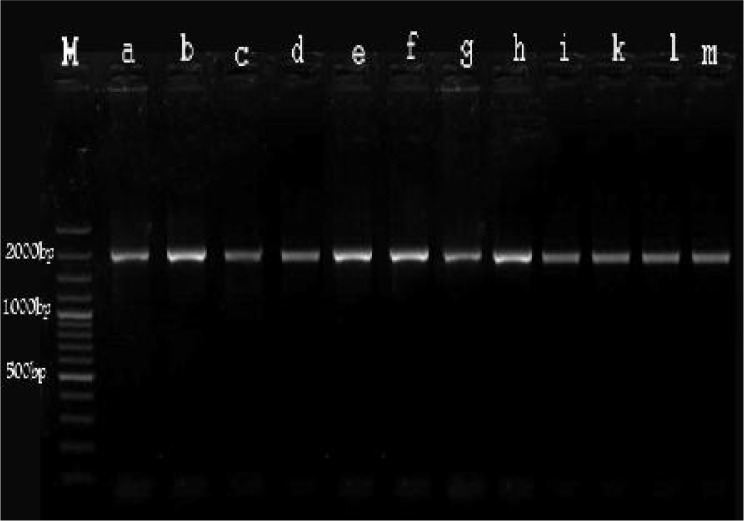
Gel electrophoresis of PCR products: M: 100 bp Marker; a-m: *Trichobilharzia regenti*

PCR products were obtained from 12 out of 45 nasal tissues (26.6%). All the products were approximately 2000bp in length (Fig. 3).

Based on sequence analysis with its5Trem primer, 9 out of 12 samples identified as *T. regenti*. They were homologous in 99% and 97% with the two sequences deposited in GenBank, one obtained from isolates from *Anas clypeata*, accession number EF094533, and the second one obtained from isolates from *Anas platyrhynchos*, accession number EF094537.

The nucleotide sequences were deposited in GenBank under following accession numbers: AB594830- AB594835.

## Discussion

Cercarial dermatitis is a known endemic parasitic disease in South and North of Iran. The infection rate of 1.1% were reported from people that had dermatitis symptoms in Khuzestan Province in the south west of Iran, and 2.4% infection with cercaria of were found in *Lymnea* snails in this area (7). In one study in North of Iran 8.5% prevalence of infection with *Trichobilharzia* and in another study 18.1% had been observed in aquatic birds (8). All of these studies focused on epidemiological aspect of cercarial dermatitis but in this study we introduced the species of *Trichobilharzia*, the most causative agent of this disease for the first time. While many studies has been done in different parts of the world on species identification of *Trichobilharzia* and so far numerous data has been published in this regard (9–14).

All samples of nasal *Schistosoma* in this work were found to be *T. regenti* except for 3 samples that failed because of low concentration of their PCR products, however, because of their approximately 2000 bp PCR band we believe that they were *T. regenti*.

Like many studies on species determination of *Trichobilharzia* our study was based on sequencing of ITS region of rDNA (10–12, 14), but there are other research suggest that D2 Domain sequences of ribosomal DNA to be a useful DNA marker for resolving species identification in avian schistosomes as well(9, 13).

In our microscopic examination we found crescent and bi-spindled shape eggs of *Trichobilharzia* in *Anas clypeata* that corresponds with the study in France, in which the eggs of *T. regenti* have shown polymorphism according to their final host species (9).

We strongly suggest that *T. regenti* is the frequent nasal schistosome of Anatid birds in North of Iran. This corresponds to the distribution of this parasite along the flyway of migratory birds, which annually migrate from Siberia and northern countries of Caspian Sea to wintering areas in southern regions of it. This research only focused on *Anas clypeata* that was the most frequent infected final host in the family of Anatidae, so further studies should be done on the other infected Anatid birds in this area in the future.
